# A Longitudinal Retrospective Observational Study on Obesity Indicators and the Risk of Impaired Fasting Glucose in Pre- and Postmenopausal Women

**DOI:** 10.3390/jcm11102795

**Published:** 2022-05-16

**Authors:** Myung Ji Nam, Hyunjin Kim, Yeon Joo Choi, Kyung-Hwan Cho, Seon Mee Kim, Yong-Kyun Roh, Kyungdo Han, Jin-Hyung Jung, Yong-Gyu Park, Joo-Hyun Park, Do-Hoon Kim

**Affiliations:** 1 Department of Family Medicine, Korea University Ansan Hospital, College of Medicine, Ansan 15355, Korea; mjintern@naver.com (M.J.N.); mytuesday89@gmail.com (H.K.); yunny23@gmail.com (Y.J.C.); 2 Department of Family Medicine, Korea University Anam Hospital, Korea University College of Medicine, Seoul 02841, Korea; chokh@korea.ac.kr; 3 Department of Family Medicine, Korea University Guro Hospital, Korea University College of Medicine, Seoul 08308, Korea; ksmpdh@korea.ac.kr; 4 Department of Family Medicine, Hallym University Medical Center, Hallym University College of Medicine, Seoul 07441, Korea; rohyk@hallym.or.kr; 5 Department of Statistics and Actuarial Science, Soongsil University, Seoul 06978, Korea; hkd917@naver.com; 6 Department of Biostatistics, Catholic University College of Medicine, Seoul 06591, Korea; jungjin115@naver.com (J.-H.J.); ygpark@catholic.ac.kr (Y.-G.P.)

**Keywords:** abdominal obesity, impaired fasting glucose, menopause

## Abstract

The impact of obesity could differ according to menopausal status since women undergo significant physiologic and metabolic changes due to menopause. We investigated the association between various major obesity indicators and the risk of impaired fasting glucose (IFG) according to menopausal status using nationally representative data. A total of 571,286 premenopausal and 519,561 postmenopausal women who underwent both Korean National Health Insurance Service (NHIS) cancer screening in 2009 and health check-ups in 2017 were analyzed. Multivariate logistic regression analyses were used to assess the effect of independent variables of body mass index (BMI), waist circumference (WC), and waist-to-height ratio (WHtR) in 2009, on dependent variable IFG in 2017. After adjusting for potential confounders, the adjusted odds ratios (ORs) and 95% confidence intervals (CIs) of developing IFG were analyzed. In the premenopausal group, the OR of obese BMI (≥25 kg/m^2^, <30 kg/m^2^) women was increased to 2.228 (95% CI: 2.139–2.321) compared to the normal BMI (≥18.5, <23 kg/m^2^) women as a reference. In the postmenopausal group, there was also a higher OR of 1.778 (95% CI: 1.715–1.843) in the obese BMI women compared to the normal group. A similar association of increasing ORs for IFG was shown in both groups when stratified by WC and WHtR. This nationwide study revealed that obesity and abdominal obesity, defined by various obesity indicators, consistently increased odds of acquiring IFG after 8 years in both pre- and postmenopausal groups, with the association being more robust in the premenopausal group. Our findings suggest that weight management and lifestyle modification may require more attention in premenopausal women.

## 1. Introduction

Obesity is known to adversely affect glucose metabolism and increase insulin resistance [1,2]. A gradual decline in insulin secretion along with a gradual increase in insulin resistance make obese subjects more vulnerable to abnormal glucose metabolism [3], which could lead to prediabetes state such as impaired fasting glucose (IFG). Several studies have revealed that an early intervention with lifestyle modification could prevent the progression of IFG to type 2 diabetes (T2DM) [4]. Moreover, recent studies show that exposure of IFG contributed to the increased risk of cardiovascular disease, and even a single exposure was associated with elevated risk of all-cause mortality [5,6].

Women who progressed from normoglycemia to IFG have greater endothelial dysfunction, more hypertension, and atherogenic risk profile than their male counterparts [7,8]. It is well known that women undergo significant physiologic and metabolic changes due to menopause. Therefore, the impact of obesity, as measured by various obesity indicators, on IFG could be different between pre- and postmenopausal populations [9,10].

Recent large population-based studies found the association between central obesity indicators, such as waist-to-height ratio (WHtR) and waist circumference (WC), and abnormal glucose metabolism to be stronger than that between total obesity indicators, such as body mass index (BMI) [11,12,13].

There are only a few reports on the difference in the impact of certain factors on the onset of disease according to menopausal status. Furthermore, the association between obesity and metabolic abnormalities such as IFG based on menopausal status requires more research. Therefore, we conducted a nationwide, large-scale cohort study to investigate the association between obesity indicators, such as BMI, WHtR, and WC and the event occurrence of IFG in pre- and postmenopausal populations. We used the Korean National Health Insurance Service (NHIS) national registration data for the diseases and health check-up data for measured values.

## 2. Materials and Methods

### 2.1. Data Source and Study Population

The NHIS is a nationwide compulsory insurance system that covers approximately 97% of the South Korean population. Although participation is not mandatory, the NHIS provides a regular standardized health check-up program every 2 years for employee subscribers, regional insurance subscribers who are regional householders, and medical aid beneficiaries. It provides an extensive medical dataset that involves health examination data and health insurance claim dataset, including diagnostic codes according to the International Classification of Diseases, 10th Revision, Clinical Modification (ICD-10-CM) codes, drug prescriptions, and demographics.

In addition to the standard health check-ups, the NHIS conducts cancer screening programs according to specific age groups. The subjects for biannual cervical cancer screening were aged ≥30 years in the year 2009. The questionnaire for cervical cancer screening included questions on menarche age, menopausal status, age, and menopause due to hysterectomy. For menopausal subjects, additional questions on the usage and duration of hormone replacement therapy were included.

**Figure 1:**
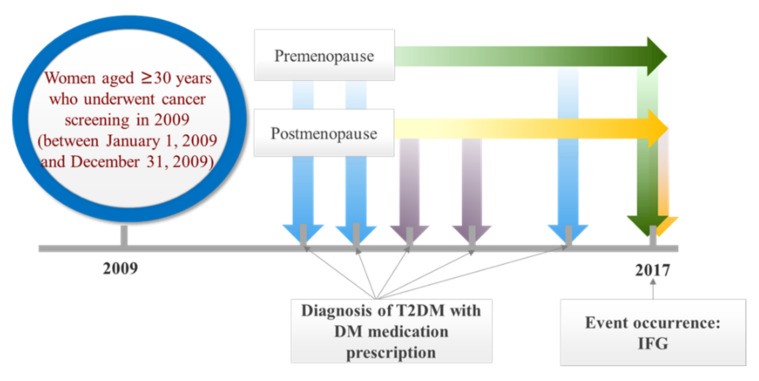
Diagram for the study design.

**Figure 2:**
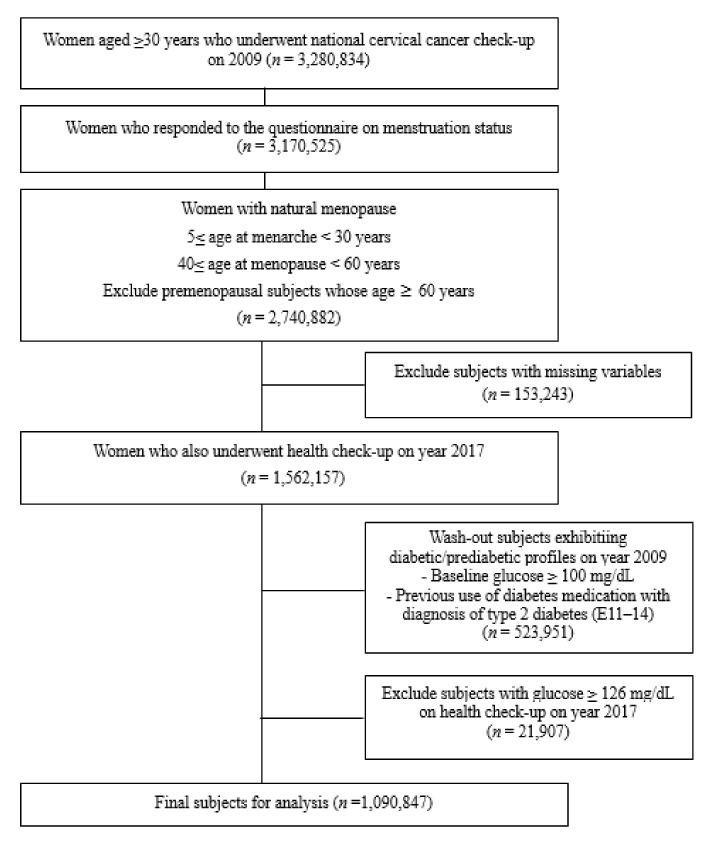
Flowchart for the selection of study population.

The design of the study is shown in Figure 1, and the flow diagram for the selection of the study population is shown in Figure 2. We selected data of women aged ≥30 years who underwent national cervical cancer check-ups in 2009 (*n* = 3,280,834). We excluded women who had menopause due to hysterectomy, and only included women with menarche age between 5 and 30 years, and menopause age between 40 and 60 years (*n* = 2,740,882). Then, we only included women who also underwent 8-year follow-up health check-up in the year 2017 (*n* = 1,562,157). Furthermore, we excluded women who exhibited diabetic or prediabetic profiles in the year 2009 (*n* = 238,991), by excluding those with baseline glucose levels ≥100 mg/dL measured during the medical check-up, and those with ≥1 claim per year for antidiabetic drug prescriptions, prescribed under ICD-10-CM codes (E10–E14) before year 2009 (index date). Finally, 571,286 premenopausal and 519,561 postmenopausal subjects were included as subjects of our analyses.

In the database, all personal identification numbers were encrypted into scrambled numbers before data processing to comply with the privacy guidelines of the Health Insurance Portability and Accountability Act. The Data Provision Review Committee of the National Health Insurance Sharing Service (NHISS) waived the need for a written informed consent as the study used de-identified data, and none of the patients were contacted. All study procedures, including ethical aspects, were approved by the Institutional Review Board (IRB) at the Korea University Ansan Hospital (IRB no. 2019AS0117) and the NHIS Big Data Steering Department (NHIS-2020-1-536).

### 2.2. Clinical and Laboratory Measurements

All participants of the NHIS health screening were required to complete self-administered questionnaires that included information on cigarette smoking and alcohol consumption, physical activity, annual income, and past medical history. Smoking status was classified according to the current cigarette smoking status, and heavy alcohol consumption was defined as the consumption of >30 g of alcohol per day. Regular physical activity was defined as follows: (1) vigorous activity for ≥20 min/day for ≥3 days/week or (2) moderate-intensity activity or walking for ≥30 min/day for ≥5 days/week. Low-income levels were defined as those eligible for medical benefits or those falling below the lower 20% of income level.

The measured values of height, weight, and WC were obtained with the participants wearing light clothing, by a trained nurse. Further, BMI was calculated as weight divided by height squared (kg/m^2^). Venous blood sampling was performed after overnight fasting to determine fasting serum glucose and total cholesterol levels.

We defined comorbidities as follows: hypertension (HTN) was defined as the presence of ≥1 claim per year of antihypertensive medication prescribed under ICD-10-CM codes of I10–13 or I15. Dyslipidemia was defined as ≥1 claim of lipid-lowering medication prescribed under ICD-10-CM code E78.

### 2.3. Obesity Indicators, Categorization, and Outcome Variables

Obesity indicators included BMI, WC, and WHtR. Each indicator was divided into four or five groups and categorized according to their characteristics.

(1) BMI was divided into five groups. Participants were categorized as underweight (<18.5 kg/m^2^), normal (≥18.5 kg/m^2^, <23 kg/m^2^), overweight (≥23 kg/m^2^, <25 kg/m^2^), obesity (≥25 kg/m^2^, <30 kg/m^2^), and severe obesity (≥30 kg/m^2^) according to the guideline strata suggested by the World Health Organization Western Pacific Region [14].
(2) WC, an indicator of abdominal obesity, was divided into five groups. A WC of ≥85 cm was considered the criterion for diagnosing abdominal obesity in Korean women [15]. The categories were 10 cm increments: <65 cm, 65–75 cm, 75–85 cm, 85–95 cm, and ≥95 cm.
(3) WHtR, which is calculated as WC/height, is also an indicator of central obesity with intra-abdominal fat [16]. The optimal WHtR cut-off value for predicting early cardiometabolic risk is ≥0.5 [17]. We divided WHtR into quartiles, where Q1 was the lowest quartile and Q4 was the highest.

The dependent variable was the event occurrence of IFG. According to the criteria of the American Diabetes Association, IFG was defined as fasting glucose level of 100 to 125 mg/dL [18]. The T2DM medication group was identified by the presence of ≥1 claim/year of anti-diabetic drug prescription under ICD-10-CM codes of E10–E14, from cancer screening examination date in the year 2009 to health check-up date in the year 2017.

### 2.4. Statistical Analyses

For baseline characteristics, we used the independent *t*-test for continuous variables and the chi-square test for categorical variables. Results were expressed as means ± standard deviations, and number (%). Multivariate logistic regression models were used to calculate the odds ratios (ORs) with 95% confidence intervals (CIs), according to BMI, WC, and WHtR in the pre- and postmenopausal groups. We adjusted for age, cigarette smoking, alcohol consumption, income level, exercise, hypertension, and dyslipidemia in Model 2 for the premenopausal group, and additionally adjusted for hormone replacement therapy usage in the postmenopausal group. For Model 3, we performed baseline glucose level adjustment in addition to the adjustments for Model 2.

Sensitivity analysis was performed to explore whether T2DM medication prescription during 8-year follow-up would affect the associations of obesity with IFG. Subgroup analyses stratified by covariates of age, smoking, drinking, low income, regular exercise, HTN, dyslipidemia, and usage of hormone-replacement-therapy category were performed in subjects with IFG for all obesity criteria. Two-sided *p*-values <0.05 were considered statistically significant. All statistical analyses were performed using SAS software version 9.4, Copyright© 2018 (SAS Institute Inc., Cary, NC, USA), and the forest plot was visualized in R Software version 3.5.1, 2018 (The R Foundation for Statistical Computing, Vienna, Austria).

## 3. Results

### 3.1. Baseline Characteristics According to the Menopausal Status

The number of IFG events were 128,465 and 148,617 in the pre- and postmenopausal groups, respectively. Table 1 shows the baseline characteristics of the study participants according to menopausal status. The average age was 43.61 ± 4.98 years in the premenopausal group and 59.45 ± 7.19 years in the postmenopausal group. The percentage of smokers, heavy alcohol drinkers, and low-income earners were higher in the premenopausal group than in the postmenopausal group. Regular exercisers were more common in the postmenopausal group. With respect to underlying diseases, the postmenopausal group had a higher percentage of subjects with hypertension and dyslipidemia. The serum fasting glucose level was 87.5 ± 7.36 mg/dL and 88.52 ± 7.22 mg/dL in the pre- and postmenopausal groups, respectively. All of these factors were significantly different between the two groups (*p* < 0.0001). We considered all of these possible confounding variables. Supplemental Table S1 shows the differences in the baseline characteristics of IFG subjects according to menopausal status.

### 3.2. Association of IFG Events with BMI and WC According to Menopausal Status

Table 2 shows the multivariate logistic analyses for the association between IFG and BMI, and WC. BMIs were divided into five groups, as mentioned in the Methods section. In the premenopausal group, the adjusted ORs (95% CI) of IFG were 0.783 (95% CI: 0.753–0.815) in underweight group (BMI < 18.5 kg/m^2^), and 2.228 (95% CI: 2.139–2.321) in severely obese group (BMI ≥ 30 kg/m^2^), compared with normal group (BMI ≥ 18.5 kg/m^2^, <23 kg/m^2^). In the postmenopausal group, the adjusted ORs (95% CI) of developing IFG were 0.846 (95% CI: 0.806–0.889) in underweight group, and 1.778 (95% CI: 1.715–1.843) in severely obese group, compared with normal group (BMI ≥ 18.5 kg/m^2^, <23 kg/m^2^). According to the results, the risk of IFG increased significantly as BMI levels increase, with the association being more robust in the premenopausal group.

For WC, five groups of 10-cm range were formed. Similar associations of increased odds of IFG event, as the WC increased were shown. The association was somewhat greater for the premenopausal group, as well. The adjusted ORs (95% CIs) in the premenopausal group for the IFG event in the <65 cm group was 0.754 (95% CI: 0.733–0.777), and 2.182 (95% CI: 2.065–2.305) in ≥95 cm group, compared with the 65–75 cm as a reference group. In the postmenopausal group, the adjusted ORs (95% CI) for the IFG according to WC was 0.825 (95% CI: 0.788–0.863) in the <65 cm group, and 1.768 (95% CI: 1.705–1.834) in ≥95 cm group. Abdominal circumference and obesity were also in positive association with IFG, in both pre- and postmenopausal groups.

### 3.3. Association between IFG Events and WHtR According to Menopausal Status

Table 3 shows the multivariate logistic analyses for association between IFG and WHtR. WHtR was divided into quartiles, with Q1 being the lowest quartile and Q4 being the highest. The association of IFG and WHtR was less profound, as there was less than two-fold increase risk of IFG of Q4 group, compared with the Q1 group as the reference. In the premenopausal group, when Q1 was set as the reference, the adjusted ORs (95% CI) of Q4 increased to 1.832 (95% CI: 1.798–1.867). For the postmenopausal group, odds of Q4 of postmenopausal group was increased to 1.518 (95% CI: 1.490–1.547), in comparison to the Q1 group. However, WHtR also showed positive associations with IFG in both groups.

### 3.4. Subgroup and Sensitivity Analyses

**Figure 3:**
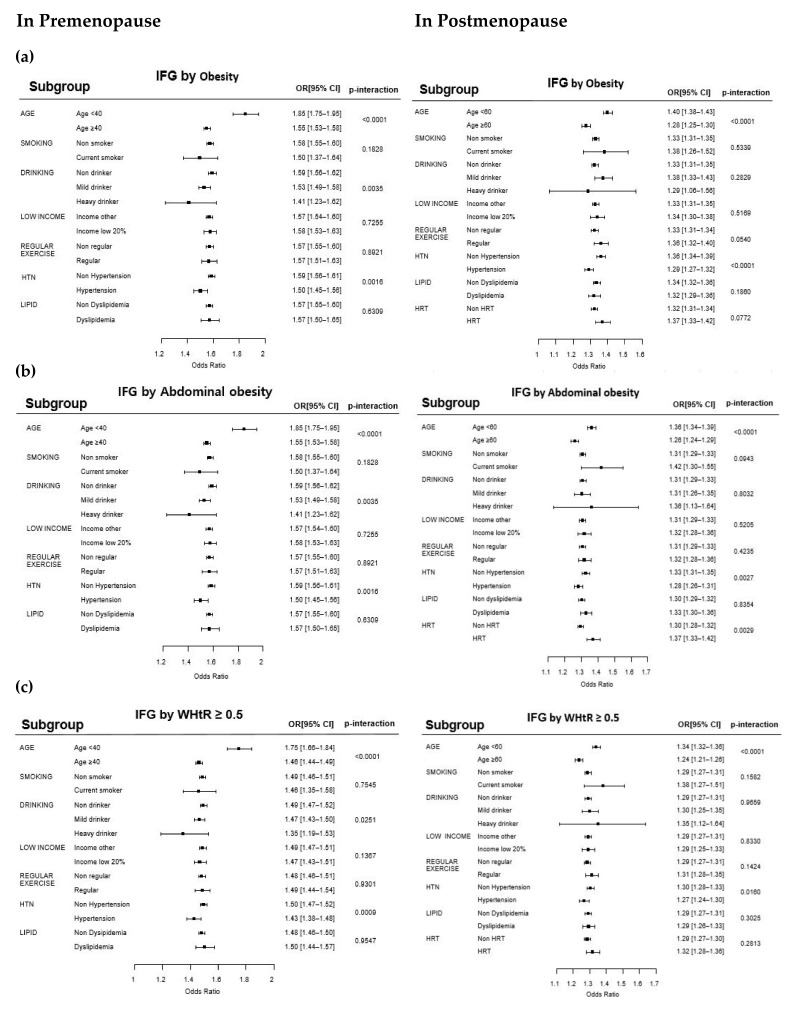
Forest plots of the odds ratios of impaired fasting glucose from various subgroup analyses in relation to different obesity criteria in the pre- and postmenopausal groups. (**a**) Subgroup analysis in obesity (BMI ≥ 25 kg/m^2^) group. (**b**) Subgroup analysis in abdominal obesity (WC ≥ 85 cm) group. (**c**) Subgroup analysis in WHtR ≥ 0.5 group. Note: Data were obtained using multivariate logistic regression models of SAS software version 9.4. The R software version 3.5.1 was employed to generate forest plots for subgroup analyses. IFG, impaired fasting glucose; OR, odds ratio; CI, confidence interval; WHtR, waist-to-height ratio; HTN, hypertension; LIPID, dyslipidemia; HRT, use of hormone replacement therapy.

Subgroup analyses were performed separately for premenopausal and postmenopausal groups, and the results are shown in Figure 3, as forest plots. We examined the findings by subgroups of age, smoking, drinking, low income, regular exercise, HTN, dyslipidemia, and usage of hormone replacement therapy. There were no changes in the main relationship between IFG event and BMI, WC, and WtHR according to subgroups. However, both younger age in premenopausal (<40 years) and postmenopausal (<60 years) groups showed significant increased interactions (*p*-interaction <0.001) to IFG event and obesity associations, and having HTN showed significant decreased interactions.

Sensitivity analyses were performed using the samples after excluding the individuals who have taken T2DM medications from cancer screening examination date in the year 2009 to health check-up date in the year 2017 (Supplemental Table S2). The positive associations between IFG events and BMI, WC, and WtHR were maintained in both premenopausal and postmenopausal groups after excluding people who have taken T2DM medication before. Except for the WtHR in the premenopausal group (*p* for interaction 0.003), there was no statistically significant interaction of T2DM medication in the association of IFG events to obesity.

### 3.5. Associations of IFG Events According to the Change of Obesity in the Premenopausal and Postmenopausal Groups

We have done multivariate logistic regression analyses to derive ORs and 95% CI for IFG event according to change in obesity (Supplemental Figure S1). Obesity change was divided in to four groups by presence or absence of obesity in first health examination date in 2009 and follow-up date in 2017 (the non-obese to non-obese group, the obese to non-obese group, the non-obese to obese group, and the obese to obese group). Interestingly, when stratified by change of obesity after 8 years, the risk of the group who changed to obesity from non-obesity was higher than the group who changed to non-obesity from obesity, and the risk of the group of maintained obesity was the highest.

## 4. Discussion

This nationwide, population-based, longitudinal, large-scale study showed that the associations between IFG and obesity and abdominal obesity, defined by various obesity indicators, were distinct in both pre- and postmenopausal women. The associations of IFG and BMI, WC, and WtHR were more pronounced in premenopausal women than in postmenopausal women. In subgroup analyses, the positive associations between IFG and obesity remained significant in all of the subgroups and younger age showed increased interactions.

Few studies on the association between obesity indicators and IFG among women have demonstrated findings similar to ours. A cross-sectional study of 712 females in China from 21 to 79 years of age, showed increased adjusted ORs of IFG for overweight (24 kg/m^2^ ≤ BMI < 28 kg/m^2^) and obesity (BMI ≥ 28 kg/m^2^) groups, with 2.43 (95% CI: 1.79–3.28) and 3.98 (95% CI: 2.64–6.00), respectively, compared with normal-weight subjects. WC also showed positive association, with adjusted ORs increased to 3.00 (95% CI: 2.18–4.13) in WC ≥ 80 cm group compared with WC < 80 cm group [19]. Another study of 265 Australian women of age 46–57 (110 pre-, 138 peri-, 17 postmenopausal) showed increasing ORs of development of IFG in groups of BMI ≥ 25.7 kg/m^2^, and decreased ORs in BMI ≤ 24.0 kg/m^2^, and even more decreased ORs in the BMI < 22.0 kg/m^2^ group compared with the BMI 24.1–25.6 kg/m^2^ group (exact values not shown, as drawn by figure) [20]. However, the study was conducted in a small population and the age range was wide.

Obesity is recognized as one of the most prominent risk factors for impaired glucose metabolism in women [21]. Moreover, maintaining a healthy weight has been of clinical importance to Asian women, as weight gain puts them at a higher risk of obtaining impaired glucose metabolism than it does women from other ethnicities [22]. Although the pathophysiological mechanism underlying this observed difference is not well known to date and requires further investigation, we hypothesized several reasons for more increased odds in the premenopausal groups in our study.

The differences in sex hormones between the two groups, accompanied by obesity, may be responsible. Among the sex hormones, estradiol is known to improve insulin sensitivity and decrease hepatic gluconeogenesis, and a deficiency of estradiol is associated with a dysregulation of glucose homeostasis in women [23,24]. It has been found that obese premenopausal women exhibited significantly lower estradiol levels than their non-obese counterparts and obese postmenopausal women exhibited significantly higher (*p* < 0.001) estradiol levels than their non-obese counterparts [25,26,27,28]. In some studies, this held true for all of the obesity indicators in our study, BMI, WC, and WtHR [25].

The difference in the site of fat accumulation might be a possible explanation of the impact of obesity in the increased risk of IFG. Premenopausal women are more prone to accumulate fat in the gluteal–femoral region, whereas postmenopausal women tend to accumulate fat in the upper body, including the abdominal area [29,30]. Increase in the accumulation of subcutaneous gluteal–femoral fat is positively associated with insulin sensitivity, and protection from fasting insulin levels [31,32]. Observational studies have suggested that WC, as compared with body weight, had a more positive association with estrogen levels, and the effect was predominant in postmenopausal women. This result was not observed in premenopausal women [33].

Additionally, overweight postmenopausal women are considered to have a higher production of endogenous estrogens by the mesenchymal adipose tissue than lean women, which could act centrally to decrease the follicle-stimulating hormone (FSH) levels [34]. FSH levels have been shown to be positively associated with serum sex hormone-binding globulin (SHBG) levels in postmenopausal women [35], due to which overweight postmenopausal women have lower SHBG levels. Increased adiposity could also induce the suppression of hepatic SHBG synthesis, [33,36] and SHBG levels in postmenopausal women are negatively correlated with visceral fat [37]. Therefore, overweight postmenopausal women also have lower SHBG levels. SHBG is known to be a strong independent marker of insulin resistance and T2DM risk, [38,39] and its level is negatively associated with the lowering of fasting plasma glucose levels [40]. Women who developed IFG have shown to have lower SHBG as well (*p* < 0.05) [20].

Our study has some limitations. First, participation in the national health examination was not mandatory, despite the fact that the examination covers health screening for all insured persons and their dependents. The results would, therefore, vary according to the socioeconomic status, which can significantly influence health behaviors. Second, although we adjusted our final results for alcohol consumption status and regular physical exercise, other important predictors of fasting glucose levels such as dietary habits and genetics could not be fully reflected in our results. Third, we have included women with menarche age between 5 and 30 years to include all subjects who started menstruation and to minimize recall bias. However, different hormone effects of a number of subjects who had early or late menarche that may affect the results cannot be excluded. Fourth, although we excluded subjects with glucose ≥126 mg/dL on the examination date to rule out subjects with a probability of diabetes, there is a chance that both people without diabetes could be included, and people with diabetes could also not be excluded. Fifth, although we have included T2DM medication groups in order to minimize bias and include individuals who went back to IFG levels after time passed, we could not omit well-controlled newly diagnosed T2DM subjects, whose fasting serum glucose levels fall into the IFG category. Sensitivity analysis was done for this limitation, and it did not change the significance of the main findings. Finally, although we used retrospective cohort longitudinal data, the study is closer to a cross-sectional study in design and, therefore, it is difficult to derive causal associations. However, since IFG is usually not recorded and included in the insurance claim data, we could only check the occurrence during biannual health check-ups. In order to compensate for these limitations of the study, we have also included ORs of IFG according to change in obesity at follow-up period as Supplemental Figure S1.

However, our study also has several strengths. To the best of our knowledge, the present study is the first to demonstrate associations between IFG and BMI, WC, and WHtR while comparing the results in pre- and postmenopausal women. The database used is a well-established longitudinal nationwide database of the general population, which contributes to the value of the results. Further studies are needed to elucidate the causal relationship and mechanism underlying the observed results.

In conclusion, our study found that the association between obesity defined by BMI and IFG was distinct, the association being more significant in the premenopausal group than in the postmenopausal group. Similar associations of increasing odds were observed with respect to the central obesity defined by WC or WHtR. Early intervention of IFG is important since it could progress to T2DM or be an independent risk of increasing CVD. Therefore, our study results suggest that obese women in premenopausal period may require more attention in weight management and early lifestyle modification.

## Figures and Tables

**Table 1:** Baseline characteristics according to menopausal status.

Characteristic	Menopausal Status		
	Premenopause (N = 571,286)	Postmenopause (N = 519,561)	*p*-Value *
Age, years	43.61 ± 4.98	59.45 ± 7.19	<0.0001
Current smoker, %	17,466 (3.06)	10,798 (2.08)	<0.0001
Drinking level			<0.0001
Non, %	405,975 (71.06)	452,426 (87.08)	
Mild to moderate, %	159,860 (27.98)	64,889 (12.49)	
Heavy (≥30 g/day), %	5451 (0.95)	2246 (0.43)	
Low income, %	131,098 (22.95)	96,937 (18.66)	<0.0001
Regular physical Exercise, %	94,787 (16.59)	102,717 (19.77)	<0.0001
Hypertension, %	58,971 (10.32)	186,985 (35.99)	<0.0001
Dyslipidemia, %	42,664 (7.47)	122,997 (23.67)	<0.0001
Use of hormone replacement therapy, %		101,320 (19.5)	
Body mass index (BMI), kg/m^2^	22.75 ± 2.88	23.83 ± 2.91	<0.0001
Waist circumference (WC), cm	74.03 ± 7.61	78.61 ± 8.19	<0.0001
Waist-to-height ratio (WHtR), WC/Height	0.47 ± 0.05	0.51 ± 0.06	<0.0001
Height, cm	157.81 ± 5.19	154.12 ± 5.47	<0.0001
Weight, kg	56.66 ± 7.62	56.63 ± 7.61	0.0538
Total cholesterol, mg/dL	188.21 ± 37.27	206.98 ± 42.23	<0.0001
Fasting serum glucose level, mg/dL	87.5 ± 7.36	88.52 ± 7.22	<0.0001
Impaired fasting glucose events, %	128,465 (22.49)	148,617 (28.60)	<0.0001
Type 2 diabetes medication, %	5415 (0.95)	18,304 (3.52)	<0.0001
Obesity (BMI ≥ 25 kg/m^2^), %	112,176 (19.64)	167,430 (32.23)	<0.0001
Abdominal Obesity (WC ≥ 85 cm), %	122,173 (21.39)	230,293 (44.32)	<0.0001
Waist-to-height ratio (WHtR) ≥ 0.5, %	143,586 (25.13)	297,321 (57.23)	<0.0001

Values are presented as means ± standard deviations or numbers (%). * *p*-values were calculated using the *t*-test for continuous variables and chi-square test for categorical variables.

**Table 2:** Multivariate logistic regression analysis of the association between impaired fasting glucose events and body mass index and waist circumference according to menopausal status.

Criteria	N	IFG Events	OR (95% CI)		
			Model 1 †	Model 2 ‡	Model 3 §
Premenopause					
BMI level					
<18.5 kg/m^2^	21,730	3069	0.704 (0.677–0.732)	0.762 (0.733–0.793)	0.783 (0.753–0.815)
18.5–23 kg/m^2^	310,377	58,758	1 (Ref.)	1 (Ref.)	1 (Ref.)
23–25 kg/m^2^	127,003	31,472	1.411 (1.389–1.433)	1.334 (1.313–1.355)	1.301 (1.280–1.321)
25–30 kg/m^2^	101,208	30,935	1.885 (1.855–1.916)	1.721 (1.693–1.75)	1.649 (1.622–1.677)
≥30 kg/m^2^	10,968	4231	2.689 (2.585–2.798)	2.381 (2.288–2.479)	2.228 (2.139–2.321)
WC					
<65 cm	42,811	6153	0.696 (0.677–0.717)	0.736 (0.716–0.758)	0.754 (0.733–0.777)
65–75 cm	281,434	54,655	1 (Ref.)	1 (Ref.)	1 (Ref.)
75–85 cm	198,267	51,298	1.448 (1.429–1.468)	1.363 (1.344–1.382)	1.326 (1.308–1.345)
85–95 cm	42,972	14,104	2.027 (1.983–2.073)	1.819 (1.779–1.861)	1.729 (1.690–1.769)
≥95 cm	5802	2255	2.638 (2.500–2.783)	2.317 (2.195–2.447)	2.182 (2.065–2.305)
Postmenopause					
BMI level					
<18.5 kg/m^2^	10,330	2148	0.814 (0.775–0.854)	0.824 (0.785–0.866)	0.846 (0.806–0.889)
18.5–23 kg/m^2^	198,297	48,387	1 (Ref.)	1 (Ref.)	1 (Ref.)
23–25 kg/m^2^	143,504	41,177	1.247 (1.228–1.266)	1.202 (1.184–1.221)	1.178 (1.160–1.197)
25–30 kg/m^2^	153,159	51,138	1.553 (1.53–1.576)	1.441 (1.419–1.462)	1.397 (1.376–1.419)
≥30 kg/m^2^	14,271	5767	2.101 (2.029–2.176)	1.856 (1.791–1.923)	1.778 (1.715–1.843)
WC					
<65 cm	12,933	2531	0.789 (0.754–0.825)	0.810 (0.774–0.847)	0.825 (0.788–0.863)
65–75 cm	148,034	34,914	1 (Ref.)	1 (Ref.)	1 (Ref.)
75–85 cm	247,113	72,161	1.336 (1.317–1.356)	1.273 (1.254–1.292)	1.247 (1.229–1.267)
85–95 cm	97,155	33,340	1.693 (1.663–1.723)	1.536 (1.508–1.565)	1.483 (1.456–1.511)
≥95 cm	14,326	5671	2.123 (2.049–2.200)	1.848 (1.782–1.916)	1.768 (1.705–1.834)

Data were expressed as ORs (95% CI). † Model 1: non-adjusted. ‡ Model 2: premenopause: adjusted for age, smoking, alcohol, income, exercise, hypertension, and dyslipidemia; postmenopause: adjusted for age, smoking, alcohol, income, exercise, hypertension, dyslipidemia, and hormone replacement therapy usage. § Model 3: adjusted for baseline glucose level in addition to Model 2. N, number; BMI, body mass index; WC, waist circumference; OR, odds ratio; CI, confidence interval.

**Table 3:** Multivariate logistic regression analysis of the association between impaired fasting glucose events and waist-to-height ratio according to menopausal status.

WHtR	N	IFG Events	OR (95% CI)		
			Model 1 †	Model 2 ‡	Model 3 §
Premenopause					
Q1	142,393	23,022	1 (Ref.)	1 (Ref.)	1 (Ref.)
Q2	143,959	29,109	1.314 (1.289–1.340)	1.253 (1.229–1.278)	1.233 (1.209–1.257)
Q3	141,348	33,517	1.612 (1.582–1.642)	1.483 (1.456–1.512)	1.438 (1.410–1.466)
Q4	143,586	42,817	2.203 (2.164–2.243)	1.926 (1.891–1.963)	1.832 (1.798–1.867)
Postmenopause					
Q1	129,586	29,644	1 (Ref.)	1 (Ref.)	1 (Ref.)
Q2	130,338	35,445	1.259 (1.237–1.282)	1.215 (1.194–1.237)	1.194 (1.172–1.216)
Q3	129,220	38,978	1.456 (1.431–1.482)	1.364 (1.340–1.389)	1.330 (1.306–1.355)
Q4	130,417	44,550	1.749 (1.719–1.780)	1.568 (1.539–1.597)	1.518 (1.490–1.547)

Data were expressed as ORs (95% CIs). † Model 1: non-adjusted. ‡ Model 2: premenopause: adjusted for age, smoking, alcohol, income, exercise, hypertension, and dyslipidemia; postmenopause: adjusted for age, smoking, alcohol, income, exercise, hypertension, dyslipidemia and hormone replacement therapy usage. § Model 3: adjusted for baseline glucose level in addition to Model 2. N, number; WHtR, waist-to-height ratio; Q, quartile; OR, odds ratio; CI, confidence interval.

## Data Availability

In the database, all personal identification numbers were encrypted into scrambled numbers before data processing to comply with the privacy guidelines of the Health Insurance Portability and Accountability Act. The data we used in this study are third-party data. According to Korean law, we are not allowed to transfer any data files to a third party. However, for researchers who meet the criteria for access to confidential data, the data are available from the Korea National Health Insurance Sharing Service Institutional Data Access/Ethics Committee (https://nhiss.nhis.or.kr/bd/ay/bdaya001iv.do, accessed on 10 March 2022). Researchers can inquire about data access to the National Health Insurance data sharing service upon approval of the Institutional Review Board of their institution. After review of the Korea National Health Insurance Sharing Service Institutional Data Access/Ethics Committee, authors are required to pay a data access fee and confirm that other researchers will be able to access the data in the same manner as the authors.

## Supplementary Materials

The following supporting information can be downloaded at: https://www.mdpi.com/article/10.3390/jcm11102795/s1, Figure S1: odds ratios (ORs) and 95% confidence intervals (CIs) for impaired fasting glucose incidence according to the four categories in the change of obesity in the premenopausal and postmenopausal groups; Table S1: baseline characteristics of impaired fasting glucose incidence subjects according to menopausal status; Table S2: odds ratios (ORs) and 95% confidence intervals (CIs) for impaired fasting glucose by obesity variables according to menopausal status. Sensitivity analysis excluding subjects with diabetes medication.
