# Supplementary material for: A Longitudinal Retrospective Observational Study on Obesity Indicators and the Risk of Impaired Fasting Glucose in Pre- and Postmenopausal Women
Source: J Clin Med. 2022 May 16;11(10):2795. doi: 10.3390/jcm11102795 (PMC9147611; doi:10.3390/jcm11102795)
Supplement: Supplementary file 1 [file jcm-11-02795-s001.zip › jcm-1656227-supplementary.pdf]

A. Premenopause

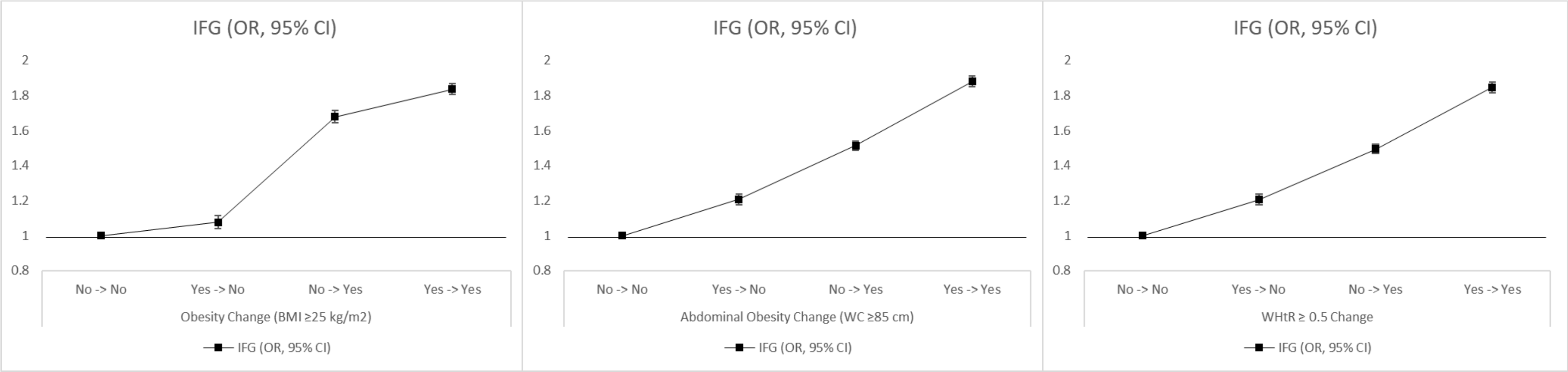

B. Postmenopause

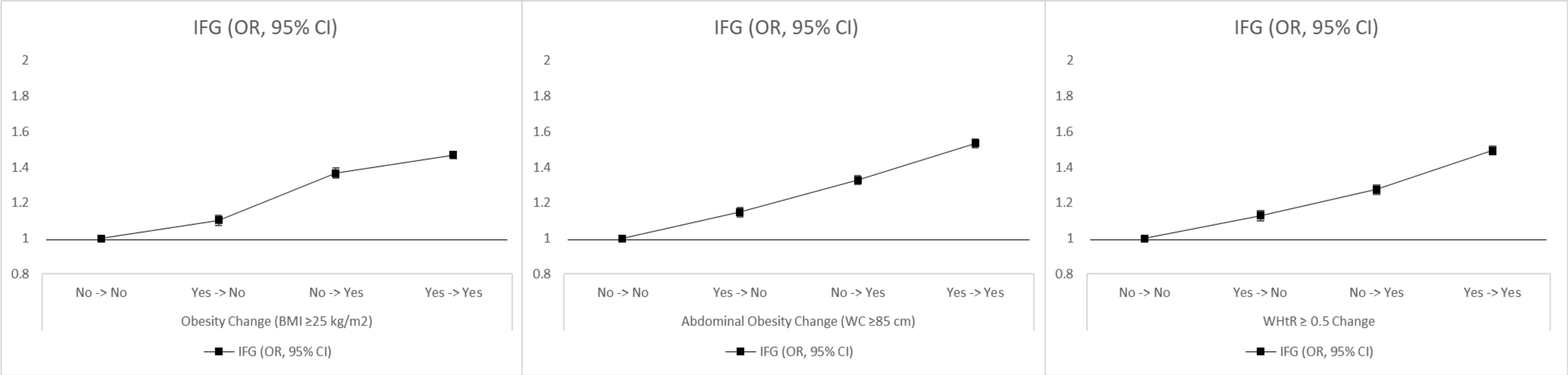

Supplemental Figure S1. Odds ratios (ORs) and 95% confidence intervals (CIs) for impaired fasting glucose incidence according to the four categories in the change of obesity in the premenopausal and postmenopausal groups. Data were obtained using multivariate logistic regression models. Premenopause: adjusted for age, smoking, alcohol, income, exercise, hypertension, dyslipidemia, baseline glucose level; postmenopause: adjusted for age, smoking, alcohol, income, exercise, hypertension, dyslipidemia, baseline glucose level, and hormone replacement therapy usage. IFG, impaired fasting glucose; OR, odds ratio; CI, confidence interval; BMI, body mass index; WC, waist circumference; WHtR, waist-to-height ratio

Supplemental Table S1. Baseline characteristics of impaired fasting glucose incidence subjects according to menopausal status

Characteristic	Menopausal status		
	Premenopause (N = 128,465)	Postmenopause (N = 148,617)	*P*-value*
Age	44.34±4.85	59.94±7.27	<0.0001
Current smoker, %	4,382 (3.41)	3,300 (2.22)	<0.0001
Drinking level			<0.0001
Non, %	89,260 (69.48)	128,691 (86.59)	
Mild to moderate, %	37,560 (29.24)	19,204 (12.92)	
Heavy (≥ 30 g/day), %	1,645 (1.28)	722 (0.49)	
Low income, %	30,197 (23.51)	27,652 (18.61)	<0.0001
Regular physical exercise, %	21,909 (17.05)	29,459 (19.82)	<0.0001
Hypertension, %	18,828 (14.66)	62,524 (42.07)	<0.0001
Dyslipidemia, %	12,327 (9.6)	38,804 (26.11)	<0.0001
Use of hormone replacement therapy, %		101,320 (19.5)	
Height, cm	157.63±5.2	154.11±5.46	<0.0001
Weight, kg	58.3±8.11	57.71±7.83	<0.0001
Total cholesterol, mg/dL	191.77±38.02	208.7±43.99	<0.0001
Fasting serum glucose level, mg/dL	89.64±6.97	90.42±6.80	<0.0001
Obesity (BMI ≥ 25 kg/m^2), %	35,166 (27.37)	56,905 (38.29)	<0.0001
Abdominal obesity, % (WC ≥ 85 cm)	37,097 (28.88)	75,562 (50.84)	<0.0001
WHtR ≥ 0.5, %	42,817 (33.33)	94,153 (63.35)	<0.0001

Values are presented as means±standard deviations or numbers (%); **P*-values were calculated using the t-test for continuous variables and chi-square test for categorical variables. BMI, body mass index; WC, waist circumference; WHtR, waist-to-height ratio.

Supplemental Table S2. Odds ratios (ORs) and 95% confidence intervals (CIs) for impaired fasting glucose by obesity variables according to menopausal status. Sensitivity analysis excluding subjects with diabetes medication.

Obesity variables		IFG OR (95% CI)	
		in Non DM Medication	in DM Medication
Premenopause			
BMI	<18.5 kg/m^2	0.764 (0.734–0.795)	0.553 (0.320–0.955)
	18.5–23 kg/m^2	1 (Ref.)	1 (Ref.)
	23–25 kg/m^2	1.325 (1.304–1.347)	1.331 (1.137–1.558)
	25–30 kg/m^2	1.681 (1.653–1.709)	1.848 (1.601–2.132)
	≥30 kg/m^2	2.241 (2.150–2.336)	2.057 (1.668–2.536)
	p for interaction	0.2418	
WC	<65 cm	0.738 (0.717–0.760)	0.649 (0.433–0.972)
	65–75 cm	1 (Ref.)	1 (Ref.)
	75–85 cm	1.347 (1.329–1.366)	1.591 (1.385–1.827)
	85–95 cm	1.758 (1.718–1.799)	2.023 (1.714–2.387)
	≥95 cm	2.180 (2.060–2.306)	2.076 (1.596–2.701)
	p for interaction	0.1017	
WHtR	Q1	1 (Ref.)	1 (Ref.)
	Q2	1.250 (1.226–1.274)	1.781 (1.385–2.291)
	Q3	1.470 (1.442–1.498)	2.386 (1.892–3.008)
	Q4	1.873 (1.838–1.909)	2.920 (2.354–3.621)
	p for interaction	0.003	
Postmenopause			
BMI	<18.5 kg/m^2	0.831 (0.791–0.873)	0.662 (0.490–0.894)
	18.5–23 kg/m^2	1 (Ref.)	1 (Ref.)
	23–25 kg/m^2	1.184 (1.166–1.203)	1.319 (1.212–1.437)
	25–30 kg/m^2	1.392 (1.370–1.414)	1.546 (1.431–1.670)
	≥30 kg/m^2	1.717 (1.654–1.783)	1.804 (1.588–2.048)
	p for interaction	0.0625	
WC	<65 cm	0.818 (0.781–0.856)	0.601 (0.427–0.846)
	65–75 cm	1 (Ref.)	1 (Ref.)
	75–85 cm	1.249 (1.230–1.268)	1.334 (1.219–1.460)
	85–95 cm	1.471 (1.443–1.500)	1.599 (1.452–1.761)
	≥95 cm	1.730 (1.665–1.797)	1.693 (1.476–1.943)
	p for interaction	0.0568	
WHtR	Q1	1 (Ref.)	1 (Ref.)
	Q2	1.201 (1.179–1.223)	1.298 (1.157–1.457)
	Q3	1.326 (1.302–1.351)	1.540 (1.381–1.718)
	Q4	1.491 (1.463–1.520)	1.698 (1.529–1.886)
	p for interaction	0.2401	

Data were obtained using multivariate logistic regression models.
Premenopause: adjusted for age, smoking, alcohol, income, exercise, hypertension, and dyslipidemia
Postmenopause: adjusted for age, smoking, alcohol, income, exercise, hypertension, dyslipidemia, and hormone replacement therapy usage.
IFG, impaired fasting glucose; OR, odds ratio; CI, confidence interval; BMI, body mass index; WC, waist circumference; WHtR, waist-to-height ratio; Q, quartile; DM, diabetes mellitus
